# Remarks on the Self-Reformation of the Medical Profession

**Published:** 1850-03

**Authors:** C. B. Coventry

**Affiliations:** Prof. of Physiology and Medical Jurisprudence in the University of Buffalo


ART. II.—*Remarks on the Self-Reformation of the Medical Profession.* By C. B. COVENTRY, M. D., Prof. of Physiology and Medical Jurisprudence in the University of Buffalo.

TO THE EDITOR OF THE BUFFALO MEDICAL JOURNAL:

DEAR SIR: Sometime since I was appointed by the Medical Society of the County of Oneida, a member of a Committee to report to the Society on the subject of Medical Reform. The accompanying desultory remarks, drawn up in a hurry, and amidst the pressure of other avocations, was the result. If you think their publication would tend to subserve the cause of *reform* about which so much has been said, they are at your service. Most respectfully, yours, . C. B. C.

GENTLEMEN: I have been invited by the kindness of the Society, and my associates, to address you on the important subject of Medical Reform. It is a broad and an interesting theme. The spirit of reform may indeed be said to be the spirit of the age. It is found pervading all departments and occupations in life. It is the very watchword in politics: every new party anxious to partake of the spoils of office, cries for reform. We hear not only of political reformers, but we have our moral reform societies, Associations for social reform, and our State Commissioners for legal reform. Efforts at reform in religion are constant. The evangelical society sends its agents to Europe, even to Italy and France, to enlighten and reform the followers of the Pope, whilst the ministers of the triple crown are laboring in our midst to reform us back to the folds of Mother Church. It would be strange, indeed, if amidst this general spirit of reformation, our own profession should remain quiet and untroubled. In Great Britain the cry for reform in the profession has been loud and long, until at length it reached the ears of royalty, or of the ministry, which is the same. It must be admitted there were many absurdities in the English laws which required reformation. Thus no man could practice in London, unless educated in particular schools. The College of Physicians, from monopolizing many privileges, had become odious. Surgeons were neither taught or examined in practice. Physicians did not study surgery, and neither studied obstetrics; indeed, it was for a long time unlawful for a surgeon to practice obstetrics, whilst the great mass of the profession, those termed general practitioners, were only educated for apothecaries, and were not permitted to charge for their services, but only for medicines furnished. This, no doubt, in many cases, led to the excessive use of medicines, often at the expense of the patient's health, as well as his pocket. Is not this the true secret of the polypharmacy of English writers on practice, which has been too often copied into the practice of our own country? These are certainly sufficient causes for reform in Great Britain. The difficulty there seems to be to satisfy the profession; the medical reform bill has been repeatedly before parliament, and has as often been met by remonstrances from some portions of the profession itself.

France, too, has caught the spirit of medical reform, and a convention was created to examine the subject and report to the government. They recommended several important alterations of the law, the most important of which was to abolish the order of "Officiers de Sante," which constituted a secondary grade of medical practitioners. I have recently seen that the minister proposes to go still further, and that no one hereafter be admitted to practice, who has not received the degree of Doctor in Medicine, and that the assumption of doctor by a person who has not received the degree, be severely punished. A reformation of this kind, which would enable the community to know who were, and who were not of the profession, is a consummation devoutly to be wished. It surely is enough that the profession should bear its own sins, but at present it has to bear the odium of the sins of every vender of drugs, or prescriber of lobelia.

Not long since it was stated that a Doctor somebody, in Michigan, had been arrested on charge of having poisoned his wife. Some of the Medical Journals expressed their regret that the profession contained such a monster; but on inquiry, it appears he is one of that class whose only claim to the title is, that they call themselves doctors. Still more recently a man by the name of Allen, styled in the papers, Dr. Allen, was committed to jail on the charge of attempting to violate the person of a young girl. The only claim, as it appears, to the title of Doctor, was that he had opened an office for vending patent medicines. If the Legislature choose to open the doors and permit every one to practice, we will not object, but if the credit of all their blunders, their vice and ignorance is to be visited on the profession, I, for one, should protest. Call them any thing but doctors, or, if they prefer this title, call us any thing else.

The subject of medical reform may, not inappropriately, be divided into three heads, viz: reform in the practice, reform in education, and the admission to the rights and privileges of the profession (if it have any), and reform, as regards the general polity and conduct of the profession, or reform of ourselves. Now, it is much pleasanter to comment upon the faults of our neighbors, and show how they could be reformed, than to examine ourselves and see where we need reformation. It is not, therefore, surprising that my associates should have selected the two former branches, and left the disagreeable task of self-examination to me. I, however, have one consolation, that we are in the happy condition of the hearer, who when the clergyman preached a sermon for his especial benefit, accosted him on going out of church, complimented his sermon very highly, and said it hit Simon B's. case exactly. Fortunately we all have the privilege of concluding the coat was cut for some one else, if it does not fit us. However important may be the subject of reform in practice, or in education, I conceive that as self reform is the first and most important of all reforms, I may flatter myself that I have the advantage in the importance of the subject, and that when once this is accomplished, all others will follow as a matter of course.

There is no observation more trite, or more true, than that in union there is strength. It is the principle of strength in our national government; it is the principle on which all voluntary associations are formed. It is, indeed, one of the first laws of nature. The cable which resists the rage of the elements, and enables the proud vessel to ride in safety, is composed of fibres which, taken separately, a child might sever. There is another maxim equally venerable, and which has the sanction of Holy Writ, "a house divided against itself cannot stand." Has it never occurred to the reformers in medicine, that these common sense maxims were as applicable to the medical profession as to the other pursuits of life? It is certain that but little can be accomplished by single and individual efforts. It is a maxim in our profession, that the first step towards successful treatment is to search for the cause or causes, and seat of the disease. Now, what is the evil which lies at the bottom of all our difficulties? I answer it is a want of public confidence, confidence not in individual members of the profession, but confidence in the profession as such. This may seem a startling proposition, but I believe it is true. I have heard an intelligent and educated clergyman observe that not one man in ten had the least confidence in the medical profession. This may be, and I hope is, an exaggeration, still, that there is too much truth, all must admit: this is the disease! But what is the cause? Is it inherent in the profession itself, or is it that the profession of medicine which was cultivated for ages before the advent of christianity, a profession, of the members of which it was said of old,

> "A good physician, skilled, our wounds to heal,
> Is more than armies to the public weal."

is, after all, entitled to no more confidence, and higher consideration? I answer, no. What then is the cause of the disease? It is the conduct of the members of the profession, and their treatment of each other! How often do we hear persons expressing regret at the loss or removal of their family physician, or when themselves about to move, a regret at going from their physician, evincing a confidence in their own physician, but a distrust in the profession. This feeling is all but universal. A confidence in their own family physician, combined with a general distrust or want of confidence in the medical profession as a whole. This is certainly the fault of ourselves; if the members are trusted as individuals, it is their own fault, if there is not the same confidence placed in them as a body. I appeal to those who hear me. Look over the broad expanse of our country in every village, town, and hamlet, who is the man, who exercises the most influence? In three cases out of four, next to the clergyman, it is the physician, who is often the confident of the family, the depository of their secrets. He is supposed to be an educated man, a reading man, and his constant intercourse with the people gives him an opportunity of knowing and consulting their feelings, possessed by no other profession. It has been justly and truly said, that there was no object in itself reasonable, so far as legislation is concerned, that they could not accomplish. Certainly no body of men could accomplish so much by concert and harmony of action, but instead of this, every man's hand is literally against his neighbor. The very confidence placed in an individual leads to a distrust of the profession, when he hears his own physician representing his brethren and rivals as fools and knaves. The result of this has been, that doctors' quarrels have become proverbial, and our noble profession trampled in the dust, a by-word and reproach. The credit which should be given to the profession as a body is monopolized by the individual, and his success is attributed to his superior skill and wisdom. A gentleman of one of the learned professions once told me, that when, on a certain occasion, riding with a medical man, he expressed a wish to, and did call on several of his professional brethren, who expressed their pleasure at seeing him. When they renewed their ride, the doctor observed, how strange this seems! we doctors never call on each other!

I am happy to know that this feeling is far from being universal. In many of our cities, and sometimes in the country, the intercourse between the members of the medical profession are as friendly and cordial as that of any profession whatever; but it is feared that there too many cases where the truth of the Doct.'s remarks would apply, that Doctors never call upon each other. But the time is approaching, if it is not already at hand, when doctors must call on each other, or our profession will cease to be worth practicing. We must not only call upon each other, but we must learn to respect each other; to stand by and aid each other in defence of ourselves. It is undoubtedly true, that medical men are too prone to comment upon and censure the practice of others, if it does not conform to their own particular views; and the most natural inference drawn by listeners and lookers on is, that one or both must necessarily be wrong. And this impression, instead of being corrected, is too frequently encouraged. It much more frequently happens that both are right, and that after all, it is only a different as to the best means of accomplishing the same object. Take, for instance, a case of inflammation of any internal organ: one man bleeds at the arm, adopting general bleeding as the quickest mode of arresting the disease; another prefers local bleeding; another thinks it best to reduce the system by the administration of saline cathartics; another will give antimony, and another prefers giving homœopathic doses, or its equivalent, nothing, and reducing the patient by the abstraction of food, and all stimuli. The same object is accomplished in all, viz., reducing the system. It is true that all cannot be best, for this depend upon the nature of the particular case. We are all prone to prefer such means as we have been accustomed to use with success; but this does not prove that some different mode may not be equally successful. The disciples of Hahnemann have frequently asserted that scarcely any two physicians of what they term the old school, agree in their practice. I deny the assertion, and venture to assert that in any ordinary disease, well educated men would not differ in one case out of ten; no, not once in a hundred. I speak advisedly on this subject, for few men have had better opportunity for knowing the the practice of the profession. Where the disease is obscure there may be difference in the diagnosis, and of course this will lead to difference in treatment; there may be some slight difference as to the best mode of accomplishing the desired object; but where they were agreed I have rarely found any difficulty in agreeing upon the means to be used. This is not the popular opinion; the general impression is, that doctors disagree among themselves, that this man bleeds, and another never bleeds, this man gives one thing and that man another thing. Is it surprising that a reflecting mind, with these impressions, should loose his confidence in the profession? I am sorry to say, that false as the impression is, and suicidal as it is, it has been too often encouraged by members of the profession. Let this be reformed altogether, and let every practitioner take pains to explain to their patients that though they may differ from another as to the precise means to be adopted, it is not a difference of principle.

You will, perhaps, ask, is it not a duty which we owe to the public to expose gross ignorance, when found, even in the profession, under the garb of ostentatious and arrogant assumption? And who would thank you for the exposure? Would knavery have one dupe the less, or ignorance loose a single victim? Our law-makers have decided that the people are competent to judge of such matters, and to interfere would be construed as interfering with their inherent rights. Exposures of this kind, are usually attributed to professional rivalry and jealousy, rather than to a sense of duty; and often proves more disastrous to the person making it, than to the person exposed. It is a short-sighted policy, to hope to elevate ourselves by depressing our neighbors. It would be much wiser to encourage, instruct, and sustain each other, so far as it could conscientiously be done, than hope to gain by exposing his mistakes and blunders to the public eye. We are all fallible, and the time may come when we ourselves may want that charity we would deny to another; better let us adopt the Golden Rule in our treatment of our Medical Brethren, and " do unto others as ye would that others should do unto you;" for this is not only " the Law and the Prophets," but it is the sum of Medical Ethics.

I have stated my belief, that a want of public confidence in the profession, as a profession, was the great evil needing correction. I have endeavored to trace the cause of this evil to its source in the misjudged policy of the members of the profession in their professional and social intercourse with, and treatment of each other. It is an old saying, that no man will be respected that does not respect himself, and it is certainly true that no profession will be respected, the members of which do not respect each other. Springing from this want of confidence in the profession are numerous evils, which spread over the land like a pestilence, and which threaten, (if not corrected,) to drive from the ranks of the Medical profession (at no distant day) every high-minded and honorable man, and reduce it to a system of quackery. Necd I refer to the host of secret and patent medicines which swarm like locusts. This must certainly be a lucrative trade in the lives and health of the people, or it would not find so many followers. Look at the thousands of dollars spent for advertising and puffing these nostrums. Who can doubt that the whole system has its origin, and aliment in the want of confidence in the profession? What sane man would resort to means of which he knows nothing, and which are as likely to kill as cure, if he felt that he could resort with confidence to the regular profession, with the full assurance that all would be done for him which his case admitted? We may, perhaps, be told that the public judge correctly and award to the profession all the confidence to which it is entitled, and that the man acts wisely who turns from the regular profession to take nostrums, of the properties of which he knows nothing. Is this indeed so? Is it true that a man who has conscientiously and honestly devoted years to the study and practice of medicine—a man whose honesty and integrity are known and admitted—after all knows nothing, and that the patient is wise to turn from his counsel and advice, and resort to means which would be much more likely to do harm than good? Some of these preparations have, no doubt, been put forth by simple-minded persons who honestly believe, that because they have succeeded in one tion of physicians which have been seized hold of by the vendor, but cure, they may be useful in others. Many have arisen from prescrip- many others have, no doubt, been got up by unprincipled persons, merely as a speculation. Look, also, at the host of new pretended systems of practice. Thompsonianism, or the self-styled Botanic Doctors, knowing as little of botany as they do of anatomy or physology; Homœopathy, Hydropathy, Uriscopy, and the Indian practice, all of which find their advocates, followers, and dupes, and all of which find their success in the want of confidence in the regular profession. It is but a few days since I was told by an intelligent man that he had frequently heard educated gentlemen say, that they believed the North American Indians had a better knowledge of Medicine than the best educated Paris physician. No person could credit this if he consulted his reason, but if he looked at the conduct of mankind, who could doubt it! The cause which we have assigned, is not the only one. Hahnemann, the founder of Homœopathy, himself a physician, began by denouncing the rest of the profession not only as ignorant, but as knaves, and as willfully deceiving the people; his disciples have followed in the same strain. The advocate of every new doctrine begins by denouncing the old. Doct. Fitch, the author of six lectures on the use of the Lungs, &c., says: "All confidence in the regular medical faculty for consumption is lost. Nobody respects them, and they do not respect themselves on this subject." Again, he says: "Nine-tenths of all that is laid down in medical books, taught in medical schools, or pursued in medical practice, for the prevention and cure of consumption, is calculated to make the disease and not to cure it." Again, he says: "In many parts of the United States, cities, towns, villages, and country are strewn with the wrecks of living men, women, and children, whilst the grave-yards conceal the decaying remains of thousands killed by Mercury." Doctor. Fitch, and his satellites, are itinerating the country, lecturing to admiring crowds, whose ears are tickled with the abuse of the regular profession, whilst their pockets are plundered. And yet, this libeler of the profession has the impudence to put M. D. to his name. It is such men—men claiming to be of the profession and yet libeling and defaming it—who are its worst enemies, and who are injuring it more than all other causes together.

A practitioner of medicine is bound by every consideration of honesty and humanity to treat his patients in the manner which he believes best; whether with lobelia, or rain water, infinitesimal doses, or no doses, wet or dry sheets. If, however, he proclaim himself a practitioner of any of these exclusive systems, I conceive that he virtually denounces and renounces the regular profession, and should be seperated from it; but if he profess to be a Homœopathist, and yet resort to Allopathic means, he practises an imposition on the public. But we are told that Homœopathy does not consist in infinitesimal doses, though they were recommended by Hahnemann, but upon the principle "similia similibus curanter," upon which it assumes to be founded; and I have known men of education, sophisticated and deluded by this pretended philosophy. I have searched the writings of Hahnemann and others, for some evidence of the truth of this maxim. I need scarcely say my search was vain. To me it seems the purest piece of hypothetical assumption I ever met, and that the idea that medicine cures diseases by creating a new disease similar, or by producing similar symptoms, is as visionary as the illusions of a mad man.

Another difficulty which has arisen out of the want of harmony and union of the profession, is the absurd, not to say oppressive legislation on the subject of medicine. The late Reviewers, after conferring with some medical men of intelligence and standing, framed an act, making it a misdemeanor to practice without a license, giving ample protection to the public and the profession, without being oppressive to any one, but striking at the root of Thompsonianism, Botanic practice, Indian doctors, and the whole host of irregular practitioners. Scarce a year, however, passed, before the Legislature was beset with petitions for its repeal. The whole host of empirics were banded to effect this object, and no man could hope for their votes, unless in favor of this measure. Did the profession rally to sustain the law and its friends in the Legislature? Not at all. The obnoxious law was repealed and the most absurd features of the old law restored. This made it unlawful to practice without a license, under a penalty of twenty dollars, but permitted any one to practice with roots and herbs, the growth of this country. Still they were not satisfied, and year after year the Legislature was beset with petitions. A very intelligent member of the committee, though in the minority, effectually killed the bill one session by calling upon the petitioners to explain the principles of their practice, which was too absurd to go down. The following is part of the answer to the question—Do you use any thing in medicines but vegetables? "One of our apothegms is that the metals and minerals are in the earth, and being extracted from the depths of the earth, have a tendency to carry all down into the earth or in the grave who use them. That the tendency of all vegetables is to spring up from the earth. Their tendency is upward. Their tendency is to invigorate, and fructify, and uphold mankind from the grave." What will future ages say, when told that this was the medical philosophy of the 19th century, and that it was on the petition of this sect that the Legislature of New York, in its wisdom, should be allowed to repeal the law prohibiting them from practising medicine. Like all new systems, Doctor T.

founds the necessity of this system on the failure of the old. He says—"that it was by the failure of this old established system of medicine to restore to health whom we are accustomed to cherish above all earthly objects, that the human mind was led to wander in this wide region of philosophy, unguided by any human theory except reason and common sense." I have been informed that the gentleman who dared, and had the honesty to expose this farago of nonsense, lost his election, the next season, through the influence of these men, and could not, from the same reason, get an election to any office, whilst to the disgrace of the medical profession, not an effort was made for his support.

Is it not surprising, in this state of things, that demagogues are found in our legislative halls, to rail against the profession, and that men who care less for their lives than the votes of their constituents, are ready to sell themselves to those who will give them their support. It is an old maxim that it is lawful to learn from the enemy, and I admit that there is one thing the profession may learn from the Thompsonians, and that is, the art of unity of action to obtain influence. It was said in a report made to the State Medical Society some years since, that there was no legislative action which was just and proper that the profession could not obtain by unity of action. No body of men have greater means at their command. Let the medical profession manifest a little of the zeal and union of their opponents; let them feel that their duty to the public, to the profession, and to themselves, is stronger than party ties, and my word for it, we should hear no more tirades against the profession in our legislative halls; or if we did, they would have no opportunity of repeating them. I have too much confidence in the profession to believe that they would ask for any thing that was wrong; but as the guardians of the public health, they are bound to protect the community against laws which are injurious; and whatever enactments are required by the public good should be demanded and enforced.

There is one point on which it appears to me the individual members of the profession might "orient," (as Mr. Mann has it,) or right themselves. Much has been said on the subject, and much censure cast on the medical schools for sending out unqualified practitioners. Did it ever occur to those who are so loud in their denunciations, that these dolts, as they are termed, are the same men that they sent from their offices as students, somewhat improved, no doubt, but still the same individuals. If you send dolts, you can hardly expect them to come out scientific and intelligent practitioners. It would be asking rather too much of the schools to expect them to furnish their pupils with brains, as well as instruction. They have no control over the pupils sent, but must do the best they can with the material sent them. The law points out the length of time, and the course to be pursued. If a student has been industrious and attentive, and faithfully complied with the law, unless he is grossly deficient, the schools are bound to pass him. If he has been idle, negligent, or is grossly incompetent, he is rejected. If the private practitioners would admit into their offices, or send to the schools, none but those who, by previous education, mental discipline, and habits of application, was qualified for the study of the profession, they would find much less cause to complain of the schools. Let this, then, be reformed altogether, and let each one for himself resolve that he will, on no consideration, receive a student into his office, until satisfied that he possesses the education and talents to qualify him for so responsible a profession.

I am happy to congratulate the Society on the prospect of better things. The recent national convention proves that the profession is awakening to the necessity of union and exertion, and although I am satisfied that a different organization will have to be adopted, before much can be accomplished, one defining more strictly the regular and irregular members of the profession, yet it is much that a beginning has been made, an association formed, around which the profession can rally. The code of Ethics promulgated, I believe, are unexceptionable; and any change of organization which may be found necessary, can hereafter be easily effected.

